# Supplementary figures and images for: The impact of cardiopulmonary exercise-derived scoring on prediction of cardio-cerebral outcome in hypertrophic cardiomyopathy
Source: PLoS One. 2022 Jan 14;17(1):e0259638. doi: 10.1371/journal.pone.0259638 (PMC8759702; doi:10.1371/journal.pone.0259638)

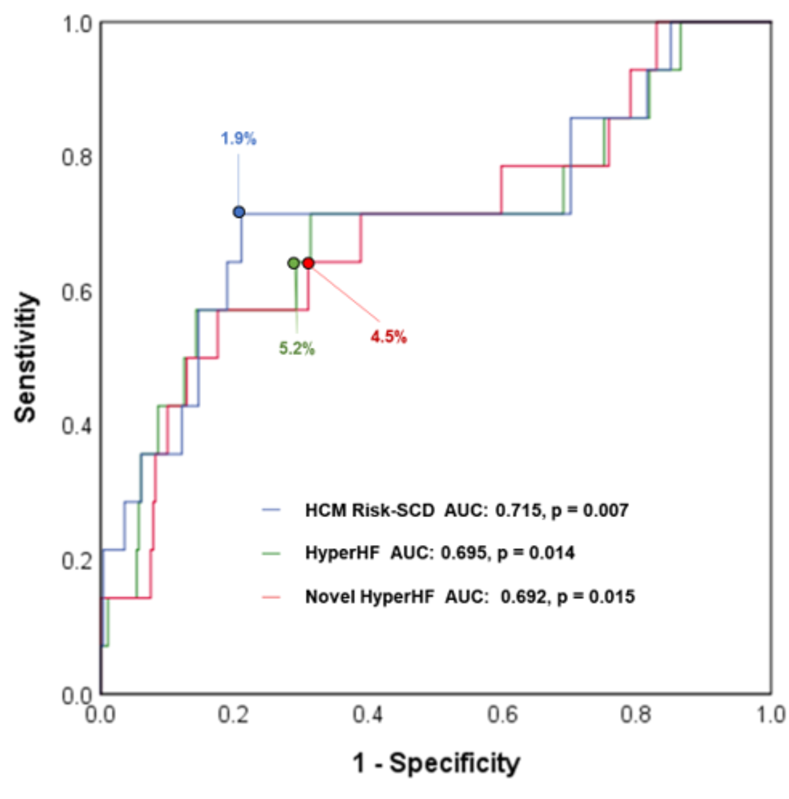

Supplement: S1 Fig — Prognostic ability of models for SCD-related events was compared among HCM Risk-SCD, HyperHF score, and Novel HyperHF score. HCM Risk-SCD score showed the highest AUC, while those of HyperHF score and Novel HyperHF were similar (0.715 vs. 0.695 vs. 0.692). The optimal cut off threshold for predicting major adverse cardiac events was based on the receiver operating characteristic curve. Each number in the curve is a cut-off value. (ROC, receiver operating characteristic; AUC, area under the curve; HCM, hypertrophic cardiomyopathy; SCD, sudden cardiac death). (TIF) [file pone.0259638.s001.tif]

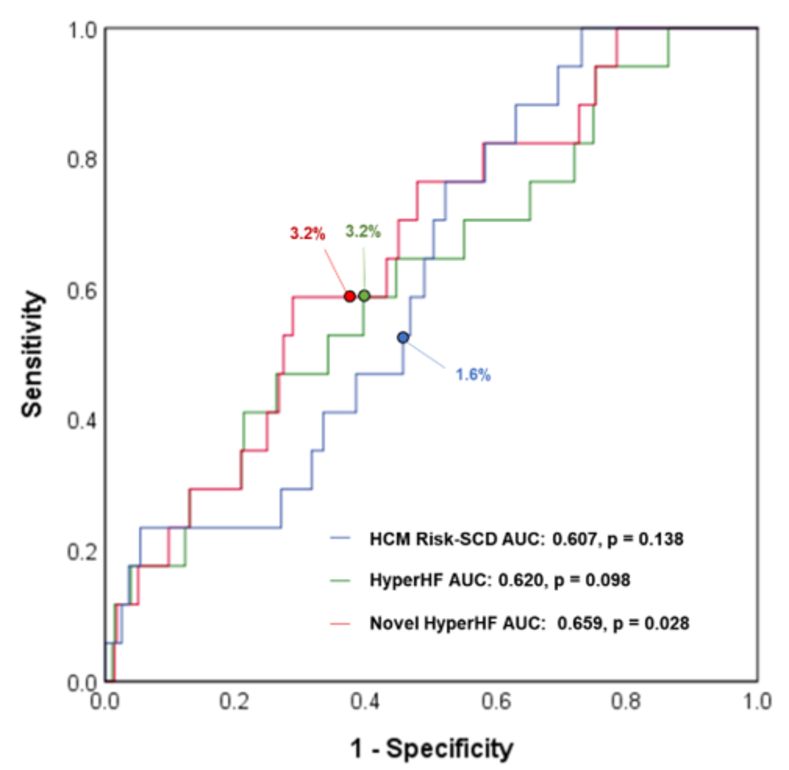

Supplement: S2 Fig — Prognostic ability of models for stroke-related events was compared among HCM Risk-SCD, HyperHF score, and Novel HyperHF score. Novel HyperHF score showed the numerically highest AUC (0.607 vs. 0.620 vs. 0.659) and had only one p value less than 0.05 (0.138 vs. 0.098 vs. 0.028). The optimal cut off for predicting major adverse cardiac events was based on the receiver operating characteristic curve. Each number in the curve is a cut off value. (ROC, receiver operating characteristic; AUC, area under the curve; HCM, hypertrophic cardiomyopathy; SCD, sudden cardiac death). (TIF) [file pone.0259638.s002.tif]

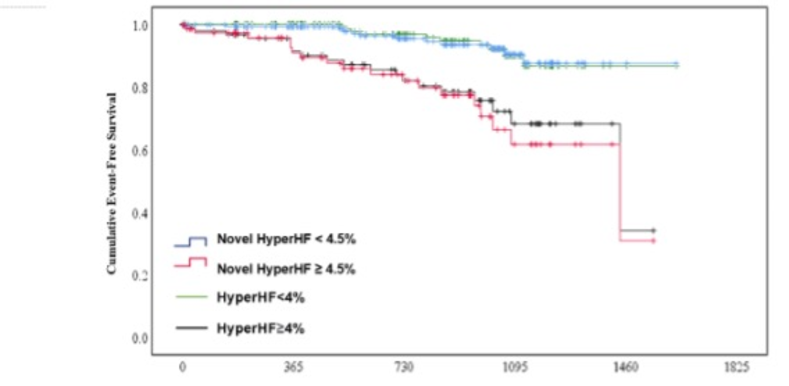

Supplement: S3 Fig — The cumulative event-free survival was compared between Novel HyperHF score and HyperHF score among total patients. Patients with higher Novel HyperHF score showed significantly lower survival after overall events during the follow-up period than patients who had higher HyperHF score. (HCM, hypertrophic cardiomyopathy; SCD, sudden cardiac death). (TIF) [file pone.0259638.s003.tif]

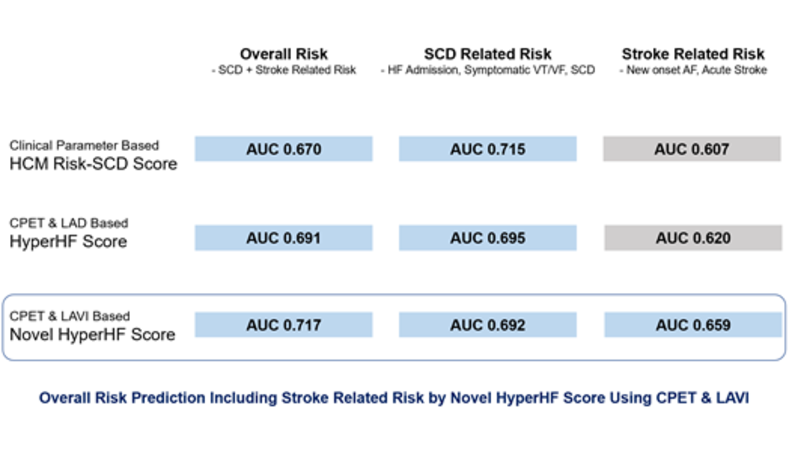

Supplement: S1 Graphical abstract — (TIF) [file pone.0259638.s007.tif]
