# Supplementary material for: The impact of cardiopulmonary exercise-derived scoring on prediction of cardio-cerebral outcome in hypertrophic cardiomyopathy
Source: PLoS One. 2022 Jan 14;17(1):e0259638. doi: 10.1371/journal.pone.0259638 (PMC8759702; doi:10.1371/journal.pone.0259638)
Supplement: S2 Table — (SCD, Sudden cardiac death; HCM, hypertrophic cardiomyopathy; PPV = positive predictive value; NPV = negative predictive value). (DOCX) [file pone.0259638.s005.docx]

Supplementary Table2. Sensitivity and Specificity, PPV, NPV of Each Cut-off point for the SCD-related Events

|  | **SCD-related Events** | | | |
| --- | --- | --- | --- | --- |
|  | Sensitivity | Specificity | PPV | NPV |
| **Novel HyperHF** | 0.643 | 0.648 | 0.083 | 0.974 |
| **HyperHF** | 0.643 | 0.690 | 0.093 | 0.975 |
| **HCM Risk-SCD** | 0.714 | 0.722 | 0.113 | 0.981 |

(SCD, Sudden cardiac death; HCM, hypertrophic cardiomyopathy; PPV=positive predictive value; NPV=negative predictive value)
