# Supplementary material for: The impact of cardiopulmonary exercise-derived scoring on prediction of cardio-cerebral outcome in hypertrophic cardiomyopathy
Source: PLoS One. 2022 Jan 14;17(1):e0259638. doi: 10.1371/journal.pone.0259638 (PMC8759702; doi:10.1371/journal.pone.0259638)
Supplement: Attachment — Submitted filename: review.docx [file pone.0259638.s008.docx]

**Major Comments**

In the present study, the aim was evaluating a scoring system predicting cardio-cerebral events in Hypertrophic Cardiomyopathy (HCM) patients using cardiopulmonary exercise testing (CPET). A total of 295 consecutive HCM patients (age 59.9±13.2, 71.2 % male) who underwent CPET was included in the present study. The previous model for SCD risk score showed fair prediction ability. However, the prediction power of Novel HyperHF showed the highest value among the models. So, the authors concluded that both conventional HCM Risk-SCD score and CPET-derived HyperHF score were useful for prediction of overall risk of SCD-related and stroke-related events in HCM. However, a novel HyperHF score using LAVI could be utilized for a better prediction power.

Despite the relevance of the issue, the manuscript needs to be improved. The Introduction Section, Materials and Methods and Discussion Section should be significantly improved. The central aim of the present study is the importance of CPET in the risk stratification model to predicting cardio-cerebral events in HCM. However, the major conclusion is that “HCM Risk-SCD score and CPET-derived HyperHF score were useful for prediction of overall risk of SCD-related and stroke-related events in HCM… a novel HyperHF score using LAVI could be utilized for a better prediction power”

**Minor Comments**

**Introduction Section**

1- The authors should include more details about the possible role of CPET assessment in stratifying overall HCM prognosis, as well as HYPertrophic Exercise-derived Risk HF (HyperHF).
2- The authors should include that the aim of the present study is about risk stratification model in HCM patients

**Materials and Methods Section**

1- The authors should include more details about the clinical characteristics of HCM patients. Were obstructive HCM patients excluded?
2- The authors should include more details about cardiopulmonary exercise testing and related parameters. Type of cycle ergometer, protocol, etc
3- The authors should include HCM Risk-SCD model in the Risk Model Verification Section

**Results Section**

1- The authors should describe all the significantly results presented in Table 2 and Table 3.

**Discussion Section**

1- What is the importance to include CPET in the risk stratification model in HCM? This is the central aim of the present study, and the authors should improve the discuss this issue.
2- In addition, the authors should improve the discuss about the Novel HyperHF using LAVI rather than LAD.
