# Supplementary material for: The impact of cardiopulmonary exercise-derived scoring on prediction of cardio-cerebral outcome in hypertrophic cardiomyopathy
Source: PLoS One. 2022 Jan 14;17(1):e0259638. doi: 10.1371/journal.pone.0259638 (PMC8759702; doi:10.1371/journal.pone.0259638)
Supplement: Attachment — Submitted filename: Rebuttal letter 2021_06 01.docx [file pone.0259638.s009.docx]

**#Reviewer 1**

**Major Comments**

In the present study, the aim was evaluating a scoring system predicting cardio-cerebral events in Hypertrophic Cardiomyopathy (HCM) patients using cardiopulmonary exercise testing (CPET). A total of 295 consecutive HCM patients (age 59.9±13.2, 71.2 % male) who underwent CPET was included in the present study. The previous model for SCD risk score showed fair prediction ability. However, the prediction power of Novel HyperHF showed the highest value among the models. So, the authors concluded that both conventional HCM Risk-SCD score and CPET-derived HyperHF score were useful for prediction of overall risk of SCD-related and stroke-related events in HCM. However, a novel HyperHF score using LAVI could be utilized for a better prediction power.

Despite the relevance of the issue, the manuscript needs to be improved. The Introduction Section, Materials and Methods and Discussion Section should be significantly improved. The central aim of the present study is the importance of CPET in the risk stratification model to predicting cardio-cerebral events in HCM. However, the major conclusion is that “HCM Risk-SCD score and CPET-derived HyperHF score were useful for prediction of overall risk of SCD-related and stroke-related events in HCM… a novel HyperHF score using LAVI could be utilized for a better prediction power

**#Answer**

Thank you for your attentive comments. I totally agree that central aim and major aim is mismatch. Actual aim of our script is comparison of scoring models and prove of usefulness of CPET as a method of predicting prognosis.

And for improvement of scripts, by CPET included as risk stratification method in 2020 AHA HCMP Guideline, I could add possible roles of CPET and HyperHF as risk stratification. And also added more details of study population, baseline characteristics, methods of CPET and details about HCM Risk-SCD model. Moreover, improve the importance of using not only CPET data as a risk stratification models but also LAVI than LAD. And for confirmative comparison of models, We proceeded Delong’s AUC comparison methods and added the results.

**Minor Comments**

**Introduction Section**

**# Comment 1**

The authors should include more details about the possible role of CPET assessment in stratifying overall HCM prognosis, as well as HYPertrophic Exercise-derived Risk HF (HyperHF).

**# Answer 1**.

Thank you for your thorough review of the script. In recent 2020 AHA Hcmp Guideline, CPET has been inserted at risk stratification. It performed to quantify the degree of functional limitation and aid in selection of patients for heart transplantation or mechanical circulatory support. And it can be helpful in differentiating HCM from other causes of ventricular hypertrophy. And it also provides noninvasive method for assessing the cardiovascular, pulmonary and skeletal muscle components of exercise performance. (1)

And the HYPERHF score represents the first attempt of an integrated risk prediction model potentially expandable to generating individualized risk estimates for HFrelated events in a contemporary HCM population. And also CPET is useful in the evaluation of HCM patients. In this context, the HYPERHF score might allow early identification of those patients at high risk of HF progression and its complications.(2, 3)

**#Comment 2**

The authors should include that the aim of the present study is about risk stratification model in HCM patients

**# Answer 2**

Thank you for your detailed comment. As you suggest, my script has vague aim. We make amends for clarify that risk stratification and benefits of CPET are our aim.

**Materials and Methods Section**

**# Comment 1**

The authors should include more details about the clinical characteristics of HCM patients. Were obstructive HCM patients excluded?

**# Answer 1**

Thank you for the detailed comment. We included 56 obstructive HCM patients, 113 non-obstructive patients and 126 Apical HCM patients.

Included subtype numbers of HCM are Obstructive 56 patients, Non-obstructive 113 patients, Apical 126 patients.

**# Comment 2**

The authors should include more details about cardiopulmonary exercise testing and related parameters. Type of cycle ergometer, protocol, etc

**# Answer 2**

We totally agree with your comment. We added details about CPET and related parameters.

**# Comment 3**

The authors should include HCM Risk-SCD model in the Risk Model Verification Section

**# Answer 3**

Thank you for the advisory comment. I added HCM Risk-SCD model at Model Verification.

**Results Section**

**# Comment 1**

The authors should describe all the significantly results presented in Table 2 and Table 3.

**# Answer 1**

Thank you for valuable advice. I added all of siginifiant results.

**Discussion Section**

**# Comment 1**

What is the importance to include CPET in the risk stratification model in HCM? This is the central aim of the present study, and the authors should improve the discuss this issue.

**# Answer 1**

We appreciate for your insightful comment. In 2020 AHA Guideline, CPET data such as Peak oxygen consumption and CO2 production predict death and provides objective data on the severity and mechanism for functional limitation. By adding these data, We can makes risk stratification model more accurate.

**# Comment 2**

In addition, the authors should improve the discuss about the Novel HyperHF using LAVI

rather than LAD.

**# Answer 2**

Thank you for thorough comments. I added benefits of using LAVI than LAD.

**Reviewer #2**

 I have read with interest your work and it surely presents new data and provide further knowledge regarding CPET and HCM.
I do have some comments and suggestions:
- Although those predictive scores are quite new and might add new prognostic information, it demands additional data. In order to investigate whether a new risk scoring model would provide complementary prognostic information to previous echocardiographic variables and/or previous risk scores, a multivariable model should be included in the result part of the paper describing the scores, differences and demonstrating this better power prediction of the new score. In this matter:
a) A kaplan-Meyer curve with both scores at the same picture with differences on prediction.
b) A table with Hazard Ratios and p values comparing both scores and some key clinical variables such as atrial diameter and volume
c) A table with the incremental value of the new score using C-Statistics

Answer A):

Thank you for thoughtful comment. We make new graph for comparison. Hyper HF score seems devided well but Novel HyperHF score devided better.

Answer B): thank you for your thoughtful comments. I added the tables as supplement for compare.

|  | Hazard Ratio | P-value |
| --- | --- | --- |
| Novel Hyper HF | 3.654 | 0.005 |
| Hyper HF | 3.035 | 0.011 |
| LAVI | 1.031 | <0.001 |
| LAD | 1.080 | 0.014 |
| PeakVO2 | 0.905 | <0.001 |
| VE/VCO2 | 1.150 | <0.001 |
| HCMRisk-SCD | 1.742 | 0.149 |

Answer C)
1.- I wonder if authors could provide additional data regarding MRI and 24-hour ECG monitoring. Some important prognostic parameters such as fibrosis and NSVT during 24-monitoring are lacking.

Answer: Thank you for your thoughtful comments. We can provide NSVT, Familiar history of SCD, Unexplained syncope history as a data parts of HCM-RiskSCD score. But Unfortunately We don’t have all patients’ data of MRI. But I agree that MRI data like fibrosis could be great prognosis factor. After much of data are collected I’ll proceed the study.

2.- It seems that LA volume index is superior to LAD and might be an explanation for the additional predictive model of the new score. It is important to demonstrate that the new score itself is the responsible for the better accuracy not LA volume index alone - another multivariable model should be enough to assess this matter.

Answer:

I appreciate to the reviewer for bringing out an important issue. We performed multivariable model analysis. As result, Novel hyperHF model is the responsible for the better accuracy itself. Here is results tables.

|  | **Univariate** | | | **Multivariate** | |
| --- | --- | --- | --- | --- | --- |
|  | P-value | CI | | P-value | CI |
| **Novel HyperHF** | <0.001 | 3.024-32.191 | 0.017 | | 1.469-46.580 |
| **LAVI** | 0.014 | 1.016-1.149 | 0.266 | | 0.987-1.049 |
| **LAD** | <0.001 | 1.014-1.049 | 0.698 | | 0.938-1.101 |
| **Gender** | 0.519 | 0.302-1.829 | 0.056 | | 0.107-1.029 |

And by inspired your advice, we performed Delong(1988) AUC comparison analysis for the new score. As a result it has no better accuracy than other scores. But in storke-related events, only p-value of AUC of Novel HyperHF below 0.05 so still in Stroke-related events new parameters cloud be used (Novel HyperHF score AUC0.659, p=0.028; Hyper HF AUC 0.620, p=0.098; HCM Risk-SCD AUC 0.607, p=0.138). Results of Delong AUC comprarisons are at below graph.

| **Cc** |  |  |  |
| --- | --- | --- | --- |
|  | HCM Risk-SCD score | HyperHF score | Novel HyperHF score |
| HCM Risk-SCD score |  | 0.6814 | 0.4479 |
| HyperHF score |  |  | 0.2017 |
| Novel HyperHF score |  |  |  |
|  |  |  |  |
| **SCD related risk** |  |  |  |
|  | HCM Risk-SCD score | HyperHF score | Novel HyperHF score |
| HCM Risk-SCD score |  | 0.8335 | 0.7990 |
| HyperHF score |  |  | 0.7904 |
| Novel HyperHF score |  |  |  |
|  |  |  |  |
| **Stroke related risk** |  |  |  |
|  | HCM Risk-SCD score | HyperHF score | Novel HyperHF score |
| HCM Risk-SCD score |  | 0.9843 | 0.6225 |
| HyperHF score |  |  | 0.0913 |
| Novel HyperHF score |  |  |  |

3.- EOV is a strong CPET parameter. Do you have data on this ?

Answer: Thank you for your valuable advice. We know there is a research that EOV could be great prognostic factor for Hypertrophic cardiomyopathy like peakVO2 and VE/VCO2. (27). Unfortunately this study is retrospective and we do not checked EOV. In next time, Using EOV as a part of predicting model might be interesting and could be better choice.

4.- Some results are included in the discussion session. It should be included in the results session and the discussion should be addressed deeply on the differences between the findings among the scores and clinical characteristics.

Answer): I totally agree with your advice. We revised results session and added discussion about differences between the findings among the scores and clinical characteristics.

1.	Ommen SR, Mital S, Burke MA, Day SM, Deswal A, Elliott P, et al. 2020 AHA/ACC Guideline for the Diagnosis and Treatment of Patients With Hypertrophic Cardiomyopathy: Executive Summary: A Report of the American College of Cardiology/American Heart Association Joint Committee on Clinical Practice Guidelines. J Am Coll Cardiol. 2020;76(25):3022-55.

2.	Magri D, Re F, Limongelli G, Agostoni P, Zachara E, Correale M, et al. Heart Failure Progression in Hypertrophic Cardiomyopathy- Possible Insights From Cardiopulmonary Exercise Testing. Circ J. 2016;80(10):2204-11.

3.	Maron BJ, Rowin EJ, Casey SA, Lesser JR, Garberich RF, McGriff DM, et al. Hypertrophic Cardiomyopathy in Children, Adolescents, and Young Adults Associated With Low Cardiovascular Mortality With Contemporary Management Strategies. Circulation. 2016;133(1):62-73.

4.	Magri D, Agostoni P, Cauti FM, Musumeci B, Egidy Assenza G, De Cecco CN, et al. Determinants of peak oxygen uptake in patients with hypertrophic cardiomyopathy: a single-center study. Intern Emerg Med. 2014;9(3):293-302.

5.	Sharma S, Elliott P, Whyte G, Jones S, Mahon N, Whipp B, et al. Utility of cardiopulmonary exercise in the assessment of clinical determinants of functional capacity in hypertrophic cardiomyopathy. Am J Cardiol. 2000;86(2):162-8.

6.	Masri A, Pierson LM, Smedira NG, Agarwal S, Lytle BW, Naji P, et al. Predictors of long-term outcomes in patients with hypertrophic cardiomyopathy undergoing cardiopulmonary stress testing and echocardiography. Am Heart J. 2015;169(5):684-92 e1.

7.	Finocchiaro G, Haddad F, Knowles JW, Caleshu C, Pavlovic A, Homburger J, et al. Cardiopulmonary responses and prognosis in hypertrophic cardiomyopathy: a potential role for comprehensive noninvasive hemodynamic assessment. JACC Heart Fail. 2015;3(5):408-18.

8.	Coats CJ, Rantell K, Bartnik A, Patel A, Mist B, McKenna WJ, et al. Cardiopulmonary Exercise Testing and Prognosis in Hypertrophic Cardiomyopathy. Circ Heart Fail. 2015;8(6):1022-31.

9.	Guttmann OP, Pavlou M, O'Mahony C, Monserrat L, Anastasakis A, Rapezzi C, et al. Prediction of thrombo-embolic risk in patients with hypertrophic cardiomyopathy (HCM Risk-CVA). Eur J Heart Fail. 2015;17(8):837-45.

10.	Magri D, Limongelli G, Re F, Agostoni P, Zachara E, Correale M, et al. Cardiopulmonary exercise test and sudden cardiac death risk in hypertrophic cardiomyopathy. Heart. 2016;102(8):602-9.

11.	Gersh BJ, Maron BJ, Bonow RO, Dearani JA, Fifer MA, Link MS, et al. 2011 ACCF/AHA guideline for the diagnosis and treatment of hypertrophic cardiomyopathy: executive summary: a report of the American College of Cardiology Foundation/American Heart Association Task Force on Practice Guidelines. Circulation. 2011;124(24):2761-96.

12.	Authors/Task Force m, Elliott PM, Anastasakis A, Borger MA, Borggrefe M, Cecchi F, et al. 2014 ESC Guidelines on diagnosis and management of hypertrophic cardiomyopathy: the Task Force for the Diagnosis and Management of Hypertrophic Cardiomyopathy of the European Society of Cardiology (ESC). Eur Heart J. 2014;35(39):2733-79.

13.	Lauer MS, Levy D, Anderson KM, Plehn JF. Is there a relationship between exercise systolic blood pressure response and left ventricular mass? The Framingham Heart Study. Ann Intern Med. 1992;116(3):203-10.

14.	Khoury S, Yarows SA, O'Brien TK, Sowers JR. Ambulatory blood pressure monitoring in a nonacademic setting. Effects of age and sex. Am J Hypertens. 1992;5(9):616-23.

15.	Wiinberg N, Hoegholm A, Christensen HR, Bang LE, Mikkelsen KL, Nielsen PE, et al. 24-h ambulatory blood pressure in 352 normal Danish subjects, related to age and gender. Am J Hypertens. 1995;8(10 Pt 1):978-86.

16.	Doonan RJ, Mutter A, Egiziano G, Gomez YH, Daskalopoulou SS. Differences in arterial stiffness at rest and after acute exercise between young men and women. Hypertens Res. 2013;36(3):226-31.

17.	Maron BJ. Clinical Course and Management of Hypertrophic Cardiomyopathy. New England Journal of Medicine. 2018;379(7):655-68.

18.	Maron BJ, Maron MS. Contemporary strategies for risk stratification and prevention of sudden death with the implantable defibrillator in hypertrophic cardiomyopathy. Heart Rhythm. 2016;13(5):1155-65.

19.	Rowin EJ, Maron BJ, Haas TS, Garberich RF, Wang W, Link MS, et al. Hypertrophic Cardiomyopathy With Left Ventricular Apical Aneurysm: Implications for Risk Stratification and Management. J Am Coll Cardiol. 2017;69(7):761-73.

20.	Maron BJ, Casey SA, Chan RH, Garberich RF, Rowin EJ, Maron MS. Independent Assessment of the European Society of Cardiology Sudden Death Risk Model for Hypertrophic Cardiomyopathy. Am J Cardiol. 2015;116(5):757-64.

21.	Leong KMW, Chow JJ, Ng FS, Falaschetti E, Qureshi N, Koa-Wing M, et al. Comparison of the Prognostic Usefulness of the European Society of Cardiology and American Heart Association/American College of Cardiology Foundation Risk Stratification Systems for Patients With Hypertrophic Cardiomyopathy. Am J Cardiol. 2018;121(3):349-55.

22.	Rowin EJ, Maron BJ, Olivotto I, Maron MS. Role of Exercise Testing in Hypertrophic Cardiomyopathy. JACC Cardiovasc Imaging. 2017;10(11):1374-86.

23.	Lester SJ, Ryan EW, Schiller NB, Foster E. Best method in clinical practice and in research studies to determine left atrial size. Am J Cardiol. 1999;84(7):829-32.

24.	Tani T, Yagi T, Kitai T, Kim K, Nakamura H, Konda T, et al. Left atrial volume predicts adverse cardiac and cerebrovascular events in patients with hypertrophic cardiomyopathy. Cardiovasc Ultrasound. 2011;9:34.

25.	Tan BYQ, Ho JSY, Sia CH, Boi Y, Foo ASM, Dalakoti M, et al. Left Atrial Volume Index Predicts New-Onset Atrial Fibrillation and Stroke Recurrence in Patients with Embolic Stroke of Undetermined Source. Cerebrovasc Dis. 2020;49(3):285-91.

26.	Fu M, Zhou D, Tang S, Zhou Y, Feng Y, Geng Q. Left atrial volume index is superior to left atrial diameter index in relation to coronary heart disease in hypertension patients with preserved left ventricular ejection fraction. Clin Exp Hypertens. 2020;42(1):1-7.

27.	Potratz M, Fox H, Rudolph V, Faber L, Dumitrescu D, Bitter T. Respiratory dyssynchrony is a predictor of prognosis in patients with hypertrophic non-obstructive cardiomyopathy. Int J Cardiol. 2021;332:105-12.
