# Supplementary material for: The impact of cardiopulmonary exercise-derived scoring on prediction of cardio-cerebral outcome in hypertrophic cardiomyopathy
Source: PLoS One. 2022 Jan 14;17(1):e0259638. doi: 10.1371/journal.pone.0259638 (PMC8759702; doi:10.1371/journal.pone.0259638)
Supplement: Attachment — Submitted filename: Response to reviewer R2.docx [file pone.0259638.s010.docx]

**#Dear Otavio R. Coelho-Filho.**

Thanks a lot for revising and comments to our manuscripts. Below sentences are major requirements of minor revision.

Before start, I revised authors one more time with their consents. If I need any paper, notice me please.

**# Comment 1.**

A rebuttal letter that responds to each point raised by the academic editor and reviewer(s). You should upload this letter as a separate file labeled 'Response to Reviewers'.

**# Answer 1**.

Thank you for comments. I separated letters which for editor, named Response to Reviewers.

**# Comment 2**
A marked-up copy of your manuscript that highlights changes made to the original version. You should upload this as a separate file labeled 'Revised Manuscript with Track Changes'.

**# Answer 2.**

I made Revised manuscript with track changes with yellow highlights.

**# Comment 3.**

An unmarked version of your revised paper without tracked changes. You should upload this as a separate file labeled 'Manuscript'.

**# Answer 3.**

I attached manuscript file. Thank you.

**# Comment 4.**
Please review your reference list to ensure that it is complete and correct. If you have cited papers that have been retracted, please include the rationale for doing so in the manuscript text, or remove these references and replace them with relevant current references. Any changes to the reference list should be mentioned in the rebuttal letter that accompanies your revised manuscript. If you need to cite a retracted article, indicate the article’s retracted status in the References list and include a citation and full reference for the retraction notice. 

**# Answer 4.**

I revised Response to reviewer files and erased duplicated references.

Thank you for critical comments and I revised it. If there is retracted reference that I missed and I committed mistakes, then please give us comments.

And in addition, 19^th^ reference is too old guidelines (2011 year) so I change it as new one (2020 guidelines)s

**# Comment 5.**
I believe that the current manuscript may improve its readability if revised by a native English speaker. I have advised the authors to have a native English speaker to proofread the manuscripts.

**# Answer 5.**

Thank you for thoughtful comments. We commissioned our manuscripts English Correction.

**# Comment 6.**
While revising your submission, please upload your figure files to the Preflight Analysis and Conversion Engine (PACE) digital diagnostic tool, [https://pacev2.apexcovantage.com/.](https://pacev2.apexcovantage.com/) PACE helps ensure that figures meet PLOS requirements. To use PACE, you must first register as a user. Registration is free. Then, login and navigate to the UPLOAD tab, where you will find detailed instructions on how to use the tool. If you encounter any issues or have any questions when using PACE, please email PLOS at [figures@plos.org](mailto:figures@plos.org). Please note that Supporting Information files do not need this step.

**# Answer 6.**

Thank you for informing us great methods for revising figures. I completed revising figures by using mentioned method.

Sincerely, Jaeman Lee, MD.
